# Supplementary material for: Dopamine blockade impairs the exploration-exploitation trade-off in rats
Source: Sci Rep. 2019 May 1;9:6770. doi: 10.1038/s41598-019-43245-z (PMC6494917; doi:10.1038/s41598-019-43245-z)
Supplement: Supplementary file 1 — Supplementary Material [file 41598_2019_43245_MOESM1_ESM.pdf]

Dopamine blockade impairs the exploration-exploitation trade-off in rats

Supporting material

François Cinotti, Virginie Fresno, Nassim Aklil, Etienne Coutureau, Benoît Girard, Alain R. Marchand, Mehdi Khamassi

Effects of positive reward prediction errors on exploration

In this section, we show mathematically that a reduction of the amplitude of reward prediction errors [62] directly translates into changes in random exploration levels in a standard Q-learning model, with or without a forgetting mechanism. In other words, we prove that inhibition of positive reward prediction errors is mathematically equivalent to changing the value of the inverse temperature and not to changing the learning rate.

The general formula for RPE is:

$$\delta_t = \begin{cases} r - Q_t(a_t) & \text{if a reward r is given} \\ 0 - Q_t(a_t) & \text{otherwise} \end{cases} \quad (12)$$

The reward prediction error (δt) is represented by the phasic dopamine signals. However, in addition, we must consider the effects of tonic dopamine (d_0). Both positive and negative RPEs are required to control learning, so the teaching signal must consist of deviations of dopamine concentration from a threshold t_0. After each rewarded step of learning, which only affects performed actions, action values are updated according to

$$Q_{t+1} = Q_t + \alpha\left(r - Q_t + d_0 - t_0\right) \quad (13)$$

However, actions values should remain constant in the absence of phasic dopamine activity. This implies that d_0-t_0 must be zero, in other words that the dopamine concentration threshold for learning must coincide and track the average level of tonic dopamine. This is consistent with the notion that RPEs adapt to the average expectation of rewards [53], [63], [64].

If we assume that the effect of dopaminergic inhibition is to reduce the original reward function, it results in a new reward function r' = g.r where g is the reduction factor (0<g<=1). We assume the factor g to be constant during the learning process, which is reasonable under pharmacological or genetic manipulations when relearning is periodically required. Dopaminergic blockade also affects tonic dopaminergic effects, by the same reduction factor g. However, because the blockade is long lasting, we expect threshold t_0 to track the new level of tonic activation, in which case we can ignore the term $g(d_0$-$t_0)$. Just in case the threshold does not have time to adapt to the new tonic dopamine level, we compared a model which takes this effect into account and found it had a worse BIC score (58936, with two extra parameters per dose) than both the model with only β free (58489 one extra parameter per dose) and the model with all three parameters optimized independently per dose (58892).

This dopamine manipulation will change the consequences of positive RPEs (when the reward is present), but not negative RPEs (in absence of reward). Importantly, it is not equivalent to reducing learning rate, which would affect the consequences of both positive and negative RPEs. In addition, this dopaminergic manipulation is assumed not to affect the revision of value for non-selected actions if a forgetting mechanism is at play (eq. 4).

We will now show by induction that under dopaminergic inhibition, the Q-values obtained at any time during learning Q’, are just scaled-down versions of the original Q-values, Q.

Starting with $Q_0=0$ and $Q_0'=0$, $Q_0'=g.Q_0$ is true.

After each rewarded step of learning which only affects performed actions

$$Q'_{t+1}=Q'_t+\alpha(r'-Q'_t)=g.Q_t+\alpha(g.r-g.Q_t)=g(Q_t+\alpha(r-Q_t))=g.Q_{t+1} \quad (14)$$

After each non-rewarded step of learning, which only affects performed actions,

$$Q'_{t+1}=Q'_t+\alpha(0-Q'_t)=g.Q_t+\alpha(0-g.Q_t)=g(Q_t+\alpha(0-Q_t))=g.Q_{t+1} \quad (15)$$

After each forgetting step, which only affects non-performed actions (if applicable)

$$Q'_{t+1}=(1-\alpha_2)Q'_t=(1-\alpha_2)g.Q_t=g.Q_{t+1} \quad (16)$$

Then plugging the scaled Q-values into the softmax function we get:

$$P(a_{t+1}=a_i)=\frac{e^{\beta Q'_{t+1}(a_i)}}{\sum_j e^{\beta Q'_{t+1}(a_j)}}=\frac{e^{\beta' Q_{t+1}(a_i)}}{\sum_j e^{\beta' Q_{t+1}(a_j)}} \quad \text{with} \quad \beta'=g.\beta \quad (17)$$

Therefore, scaling down the reward function by a factor g with dopaminergic inhibition is formally equivalent to reducing the inverse temperature by the same factor g. Noticeably, although Q-values (including asymptotic values) are reduced by dopaminergic inhibition, this effect cannot be mimicked by a simple change in learning rate. Indeed, changes in learning rate do not affect asymptotic values for a constant reward function ($\delta_t=0$ if and only if $Q_t=r$).

Supplementary Table 1. Coefficients and adjusted R^2 of the linear model between predicted and experimental action probabilities. For each block, we labeled the three possible actions as either the target (action 1 in **Supplementary Fig. 3**), the previous target (action 2; for the very first block of the session, the target of the last block was used so as to ensure all lever-label combinations were equally represented) or the remaining lever (action 3). For each rat (n=23), we then averaged the experimental probabilities of each action for bins of four trials per block, and compared them to the corresponding average theoretical probabilities as determined by current Q-values plugged into the softmax function by fitting a linear model without an intercept (experimental probabilities = b1 * softmax probabilities). This procedure was applied to separate doses, separate risk levels and to the entire experiment as reported in the table and we always found b1 very close to 1 and very good R^2 demonstrating a very good correspondence between the two.

		Risk					
		LR		HR		All	
		b_1	Adjusted R^2	b_1	Adjusted R^2	b_1	Adjusted R^2
**Flupenthixol Dose (mg/kg)**	**0**	0.99	0.98	1	0.95	0.99	0.98
	**0.1**	0.98	0.98	1	0.96	0.99	0.99
	**0.2**	0.99	0.97	1	0.97	0.99	0.99
	**0.3**	1	0.95	1	0.94	1	0.98
	**All**	0.99	0.97	1	0.95	0.99	0.98

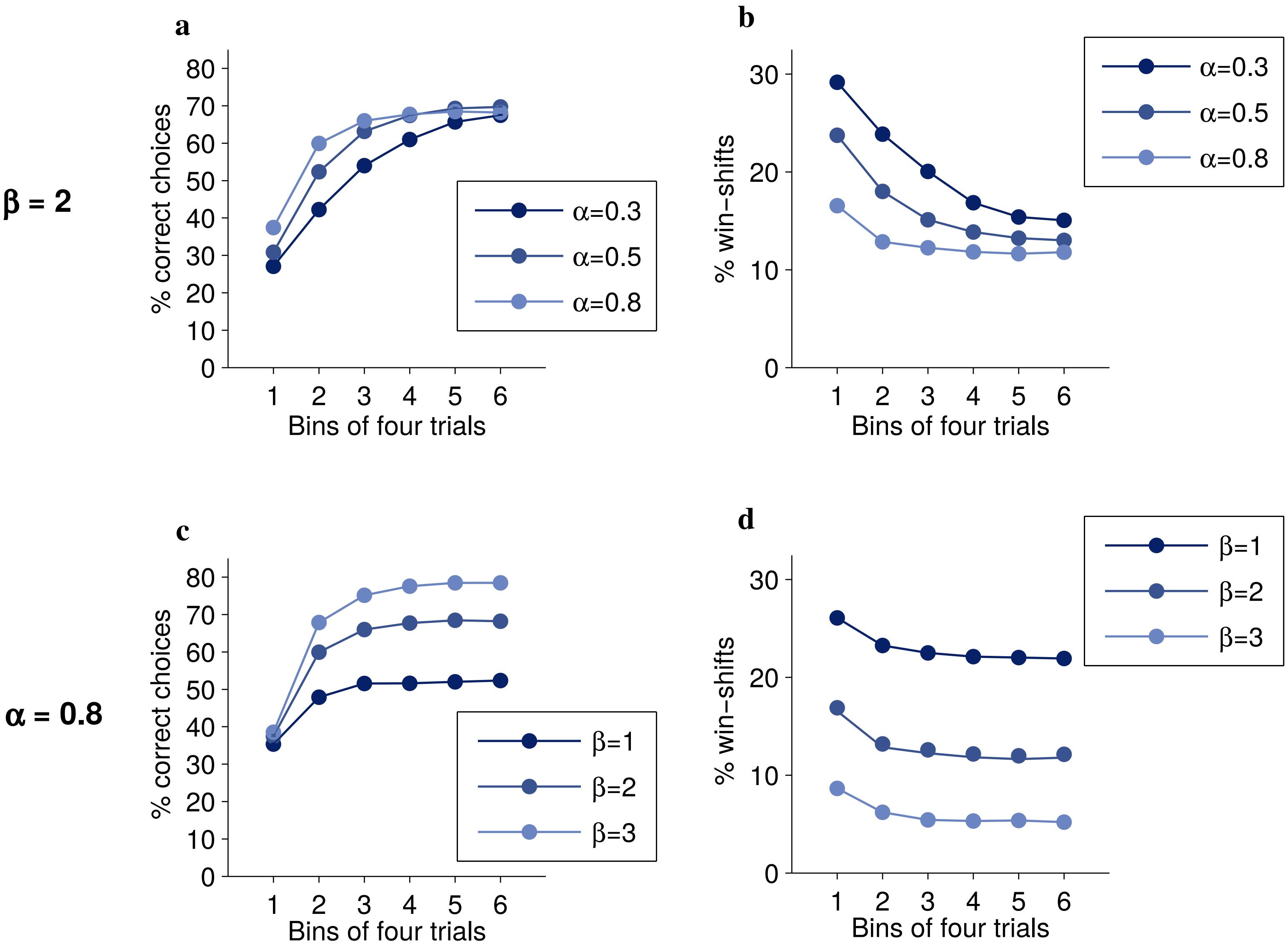

Supplementary Figure 1. Illustration of the effects of varying either the learning rate or the exploration parameter on performance and win-shift curves based on simulations of the standard Q-learning model in low risk blocks. The range of parameters was chosen for illustrative purposes and does not reflect values actually found when the model was optimized on the experimental data. **(a)** Under different learning rates, performance curves initially increase at different rates but end up converging to similar asymptotic levels. **(b)** Win-shift curves start at different levels and decrease at different rates and eventually converge. **(c)** Under different exploration rates, performance curves converge toward different asymptotes. **(d)** Win-shift curves lie parallel to one another without ever converging.

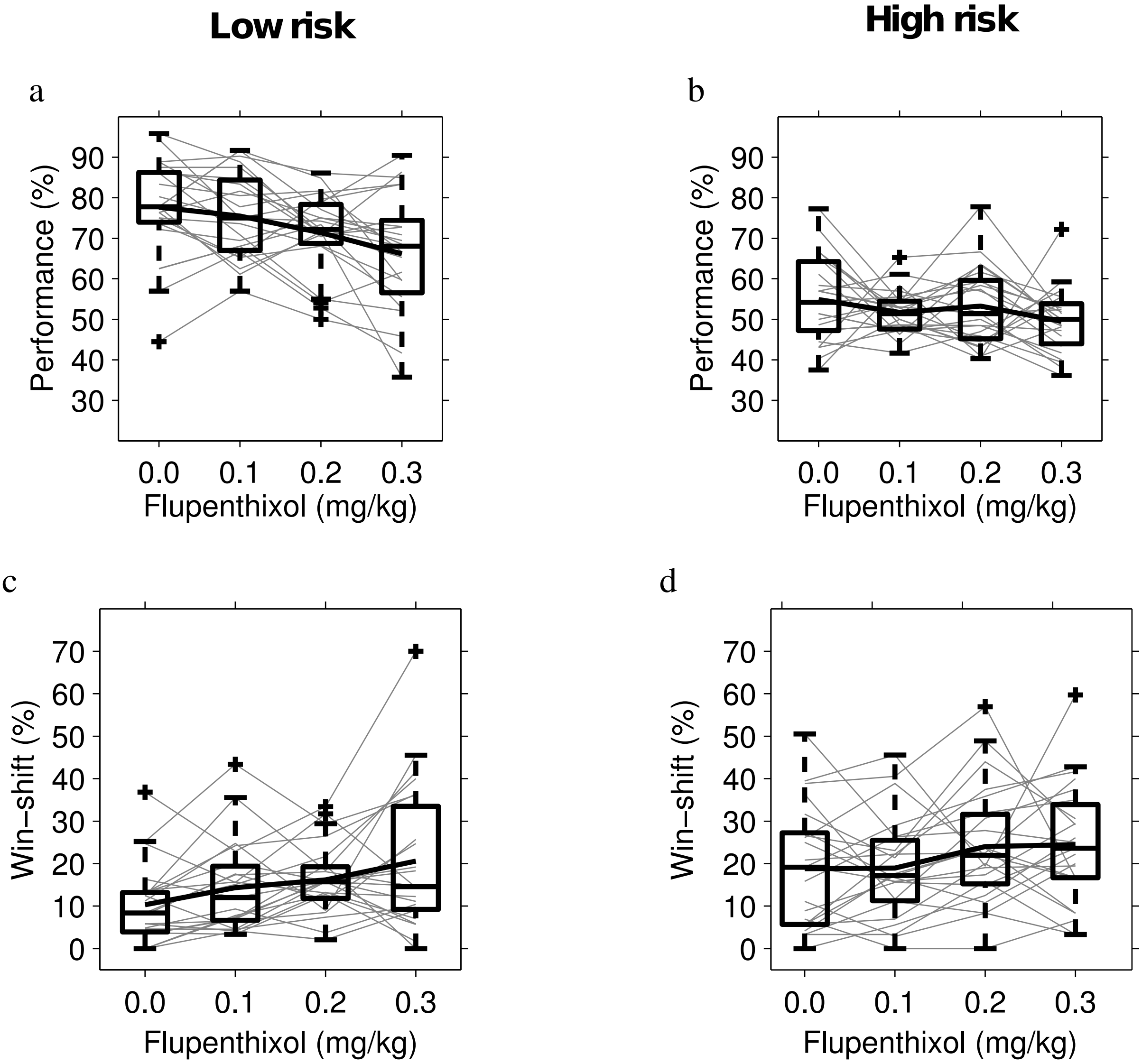

Supplementary Figure 2. Experimental effects of flupenthixol on final performance and win-shift levels. Gray lines represent individual animals, boxes show the median, interquartile range (box edges) and most extreme data points not considered as outliers (whiskers), and crosses are suspected outliers. Bold lines represent the mean.

(a) & **(b)** Effect of flupenthixol on average performance levels in the last six trials of low and high risk blocks. A significant negative effect of flupenthixol appears ($F(3,66)=8.85$, $p<0.0001$) and there is no interaction with risk ($F(3,66)=1.96$, $p=0.13$). Post hoc t-tests revealed that average performance under 0.3 mg/kg was significantly smaller than all other doses (highest $p=0.020$) and that average performance under 0.2 mg/kg was significantly worse than under 0 mg/kg ($p=0.027$). **(c)** & **(d)** Effect of flupenthixol on average win-shift in the last six trials of low and high risk blocks. A significant dose effect was detected ($F(3,66)=8.76$, $p<0.0001$) without any interaction with risk ($F(3,66)=0.77$, $p=0.51$). Post hoc t-tests showed that average win-shift increased for 0.3 mg/kg of flupenthixol when compared to 0 and 0.1 mg/kg (highest p=0.0017= and for 0.2 mg/kg when compared to 0 mg/kg ($p=0.0021$).

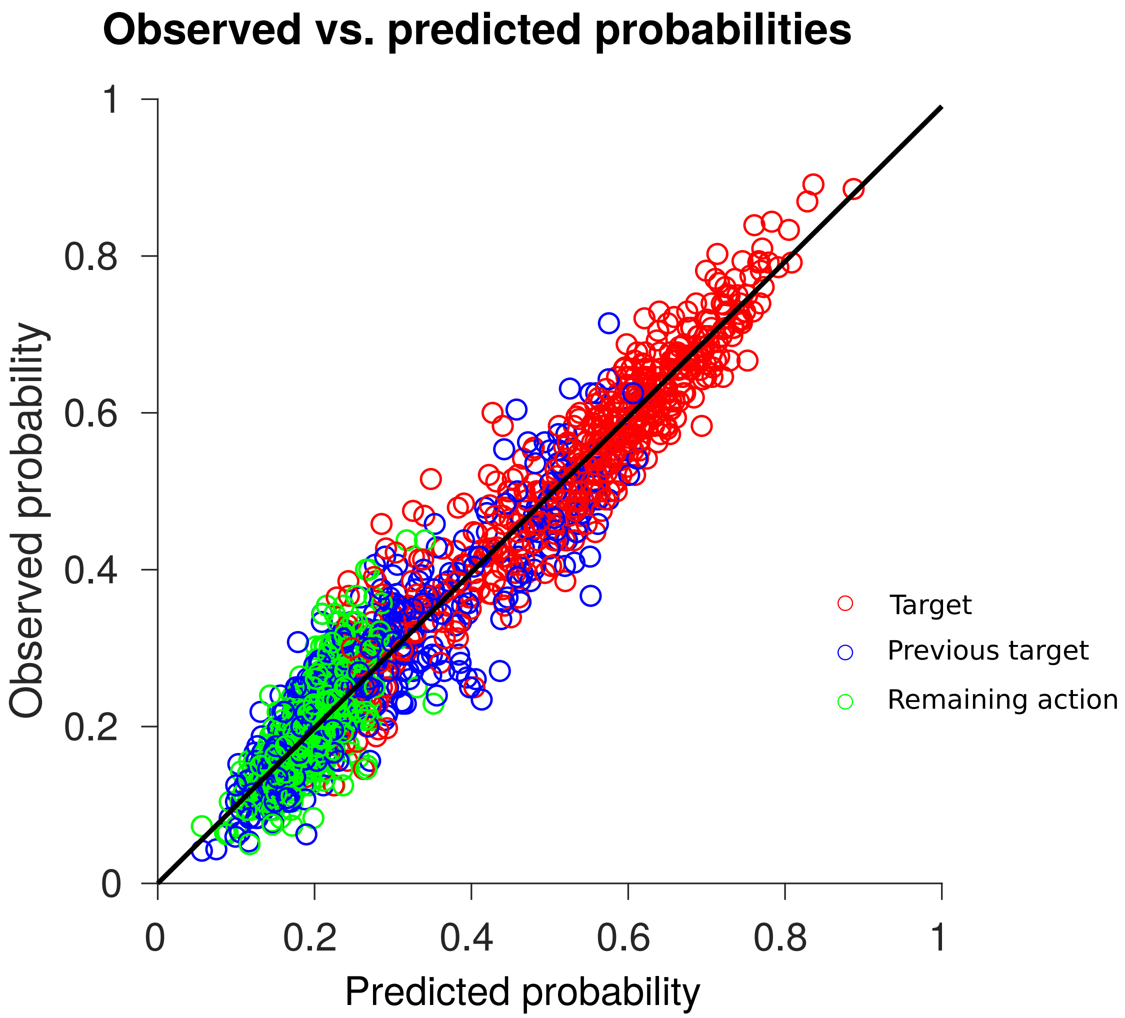

Supplementary Figure 3. Relationship between predicted and observed probabilities of the three possible actions within a block. Predicted probability is given by the softmax function averaged in a given bin of four trials within a block. Observed probability is based on the number of times this specific action was chosen in this bin. Each data point represents the average of blocks for a given combination of rat, bin, risk level, dose and response type. Response types are identified by colors representing correct (target, red), previously correct but now incorrect (previous target, blue) or other actions (green). The same linear regression curve (y = 0.99*x is shown in black) fits all three response types.

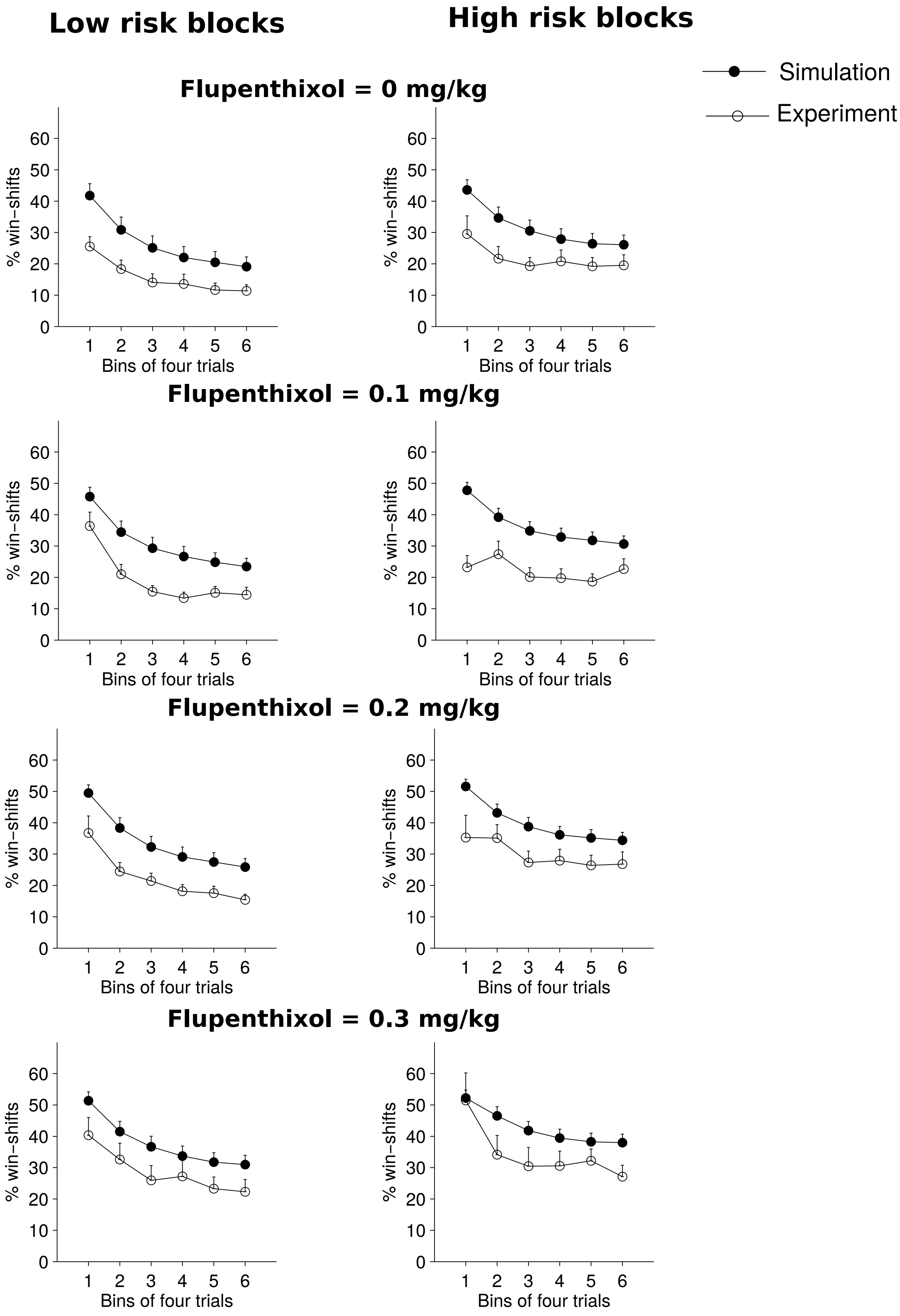

Supplementary Figure 4: Win-shift (mean + s.e.m.) of simulations of the standard Q-learning model plotted against the experimental data for the different risk and pharmacological conditions. Contrary to the forgetting model (see **Supplementary Fig. 5**), this model was unable to reproduce this key aspect of behaviour and shifted from correct rewarded actions far more frequently than the subjects.

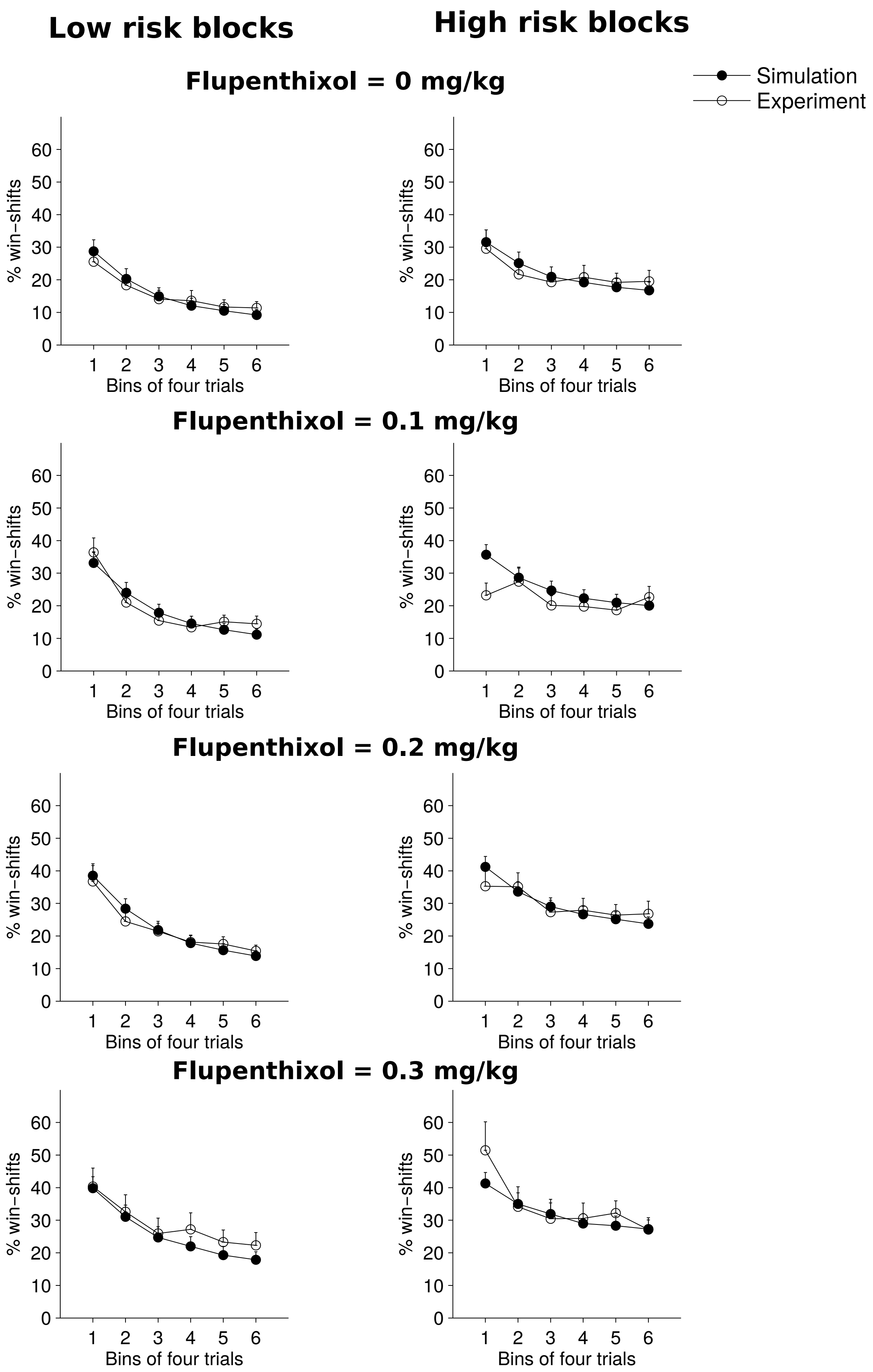

Supplementary Figure 5: Win-shift (mean + s.e.m.) of simulations of the Q-learning model extended with a forgetting mechanism plotted against the experimental data for different doses of flupenthixol and both risk levels. This figure is in fact a different representation of **Fig. 2** for comparison with **Supplementary Fig. 4**.

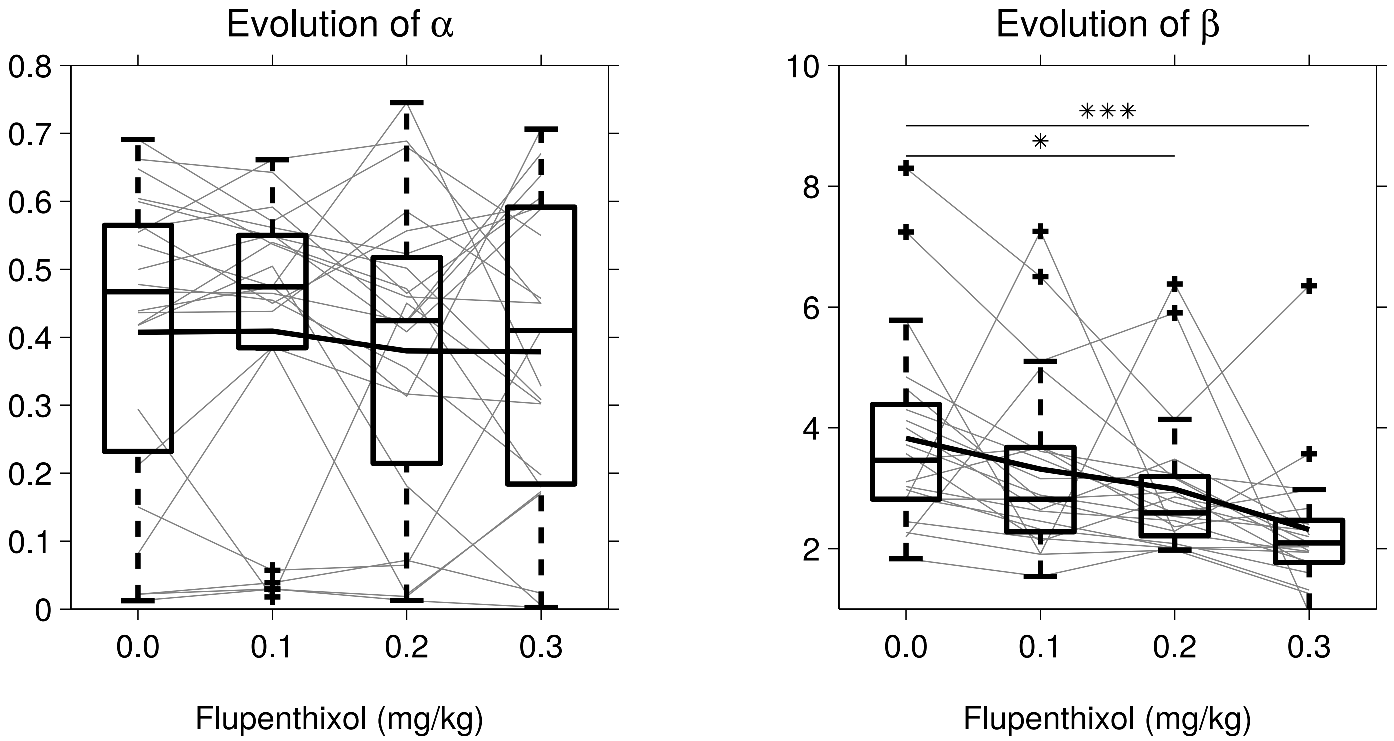

Supplementary Figure 6. Variations of the parameters of the standard Q-learning model. Gray lines connect parameter values of a same individual. Box plots of median, interquartile and furthest values not considered as outliers represented as crosses. Bold lines plot average parameter values. As with the extended model, there is no significant dose effect on α (Friedman Anova test: $\chi^2(3) = 1.90$, $p = 0.59$) whereas β is significantly affected ($\chi^2(3) = 23.5$, $p < 0.0001$).

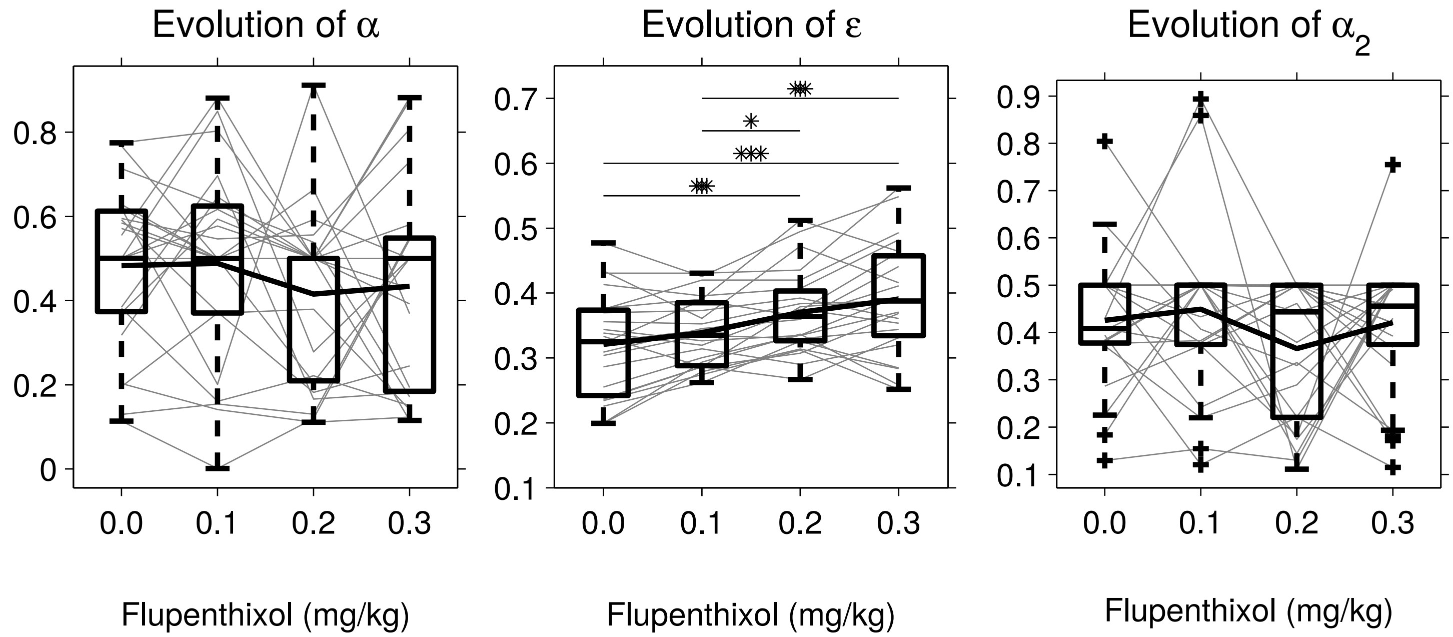

Supplementary Figure 7. Variations of the parameters of the ε-greedy Q-learning model. Gray lines plot the parameter variations of a single individual while bold line plots average parameter values. Box plot of median, interquartile and extreme values not considered as outliers repreented as crosses. As with the softmax version of this model (**Fig. 5**), the only parameter affected by flupenthixol is the one responsible for controlling exploration, ε (Friedman Anova test: $\chi^2(3)= 34.7$, $p<0.0001$).

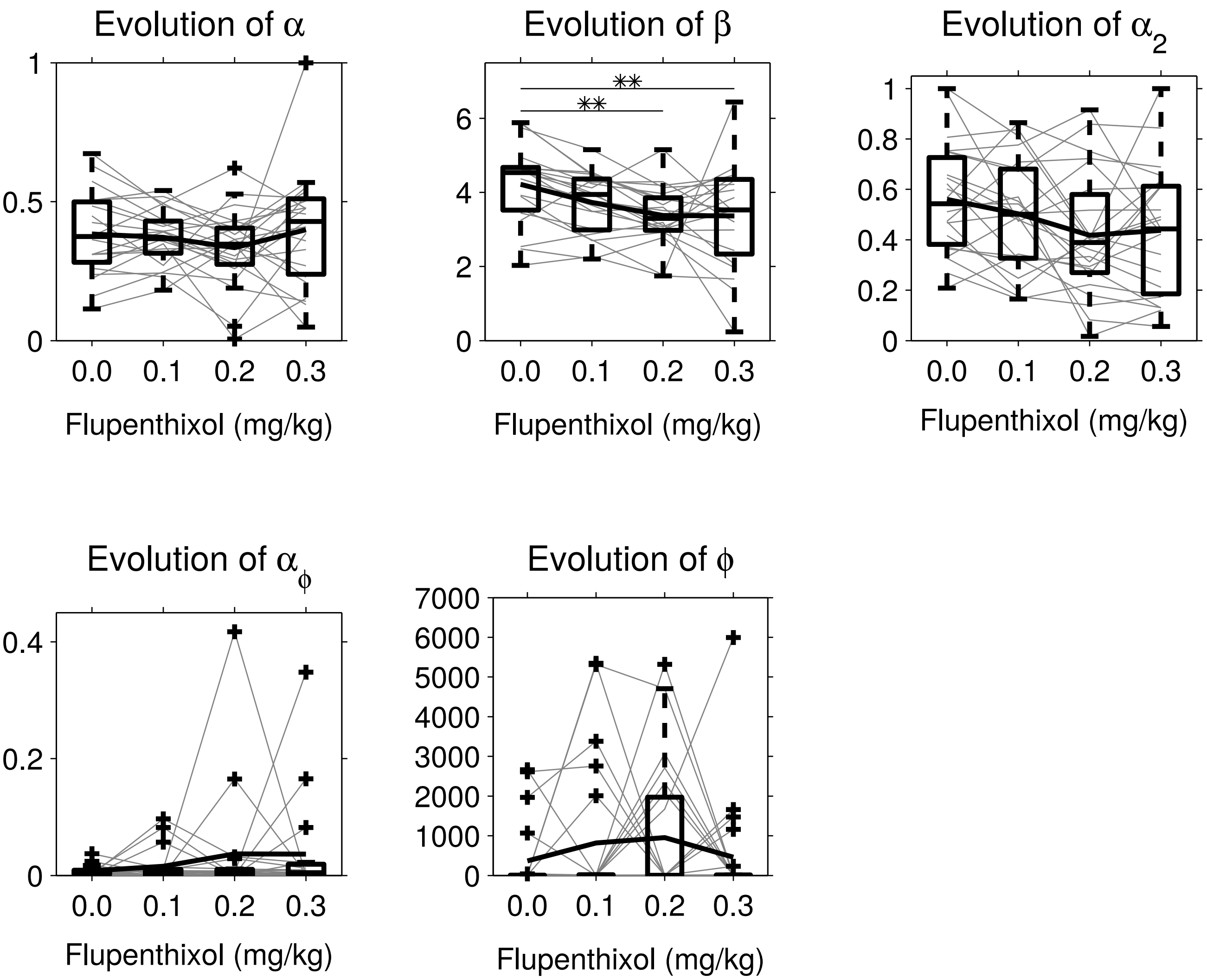

Supplementary Figure 8. Variations of the parameters of the uncertainty bonus model. Grey lines represent single individuals and bold lines the average parameter variation. Box plots of median, interquartile and most extreme values not considered as outliers. As with previous models, the only parameter affected by flupenthixol is β (Friedman Anova test: $\chi^2(3) = 16.3$, $p = 0.0010$) which controls random exploration. Exploration targeted at uncertain options is not affected.

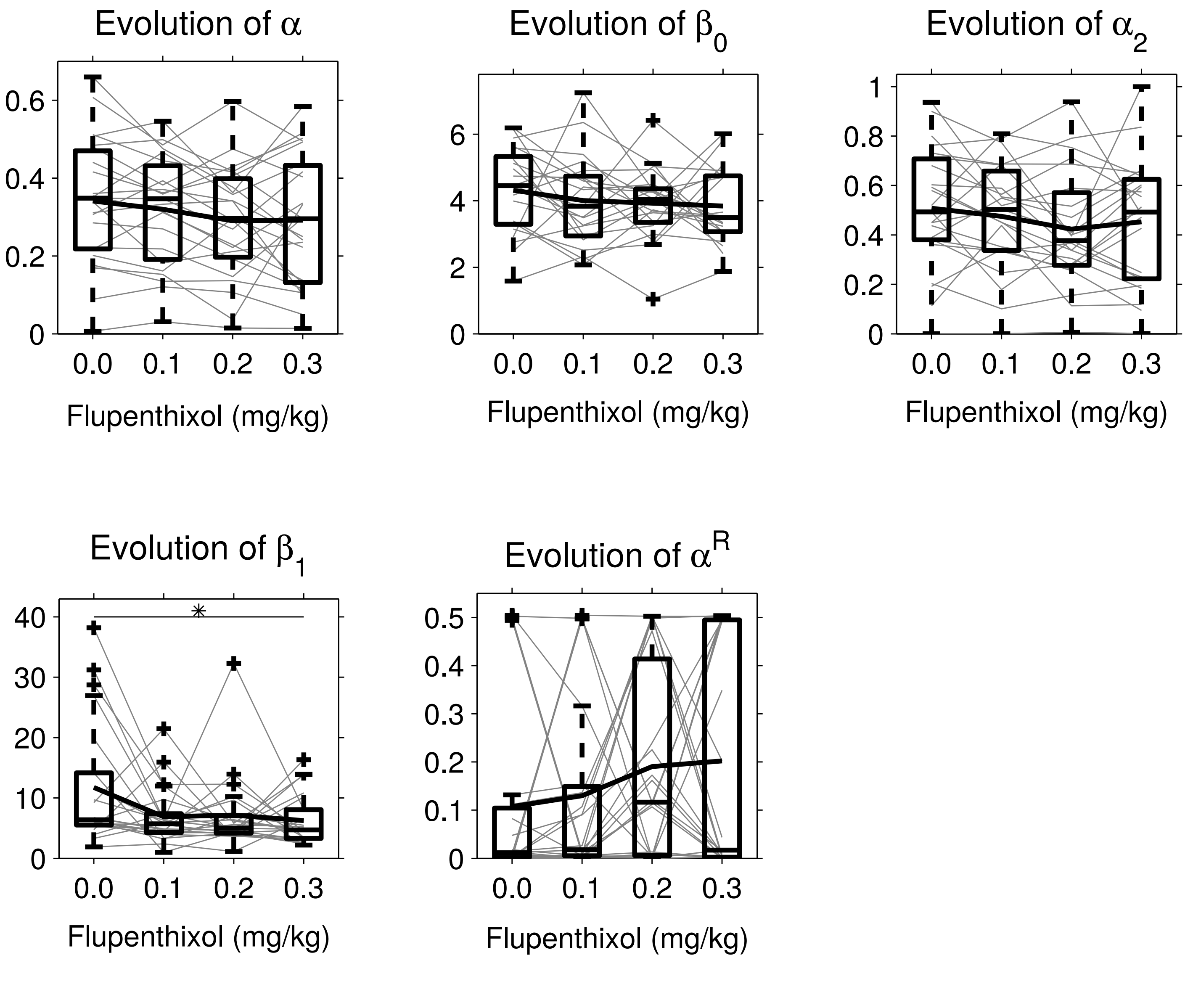

Supplementary Figure 9. Variations of the parameters of the meta-learning model. Grey lines correspond to individual rats while the bold lines represent the average of parameter values. Box plots of median, interquartile and most extreme values which are not outliers. The only parameter affected by flupenthixol is β_1 although the statistical effect is in fact quite modest (Friedman Anova test: $\chi^2(3)= 9.3$, $p=0.026$).
